# Significance of prophylactic intra-abdominal drain placement after laparoscopic distal gastrectomy for gastric cancer

**DOI:** 10.1186/s12957-015-0591-9

**Published:** 2015-05-12

**Authors:** Noriyuki Hirahara, Takeshi Matsubara, Hikota Hayashi, Kiyoe Takai, Yusuke Fujii, Yoshitsugu Tajima

**Affiliations:** Department of Digestive and General Surgery, Shimane University Faculty of Medicine, 89-1 Enya-cho, Izumo, Shimane 693-8501 Japan

**Keywords:** Gastric cancer, Laparoscopic distal gastrectomy, Prophylactic drainage, Postoperative complication

## Abstract

**Background:**

Unnecessary intra-abdominal drain insertion must be avoided, but little is known about the value of prophylactic drainage following laparoscopic distal gastrectomy (LDG). In this study, we investigated the significance of prophylactic drain placement after LDG for gastric cancer.

**Methods:**

Seventy-eight consecutive patients with gastric cancer who underwent LDG in our department were retrospectively analyzed. The patients were divided into two groups according to the insertion of a prophylactic intra-abdominal drain following LDG. The ‘drain group’ comprised 45 patients with routine use of a prophylactic intra-abdominal drain, and the ‘no-drain group’ comprised 33 patients who did not undergo placement of an intra-abdominal drain.

**Results:**

There were no significant differences in terms of the mean age of the patients, male/female ratio, body mass index, and concurrent diseases between the drain group and the no-drain group. In addition, there were no significant differences in the tumor location, tumor diameter, depth of the tumor, nodal metastasis, and tumor stage between the two groups.

All patients in each group were successfully treated with R0 surgery, and no patient required conversion to open surgery. Surgery-related factors, including lymph node dissection and operative time, were similar in the drain group and the no-drain group.

A comparison of the amount of intraoperative blood loss between patients with and without postoperative complications revealed that patients who experienced postoperative complications had a significantly larger amount of blood loss than those without postoperative complications.

A comparison of operative times between patients with and without surgery-related postoperative local complications revealed that patients who experienced surgery-related postoperative local complications had a significantly longer operative time than those without surgery-related postoperative local complications. Analysis of operative times in each group revealed that patients with surgery-related postoperative local complications had a significantly longer operative time than those without surgery-related postoperative local complications in the no-drain group.

**Conclusions:**

Intraoperative factors such as the operative time and the amount of intraoperative blood loss affected the occurrence of postoperative complications following LDG. A prophylactic drain may thus be useful in patients at higher risk and in those with a longer operative time or massive intraoperative bleeding.

## Background

The Guideline for Prevention of Surgical Site Infection (SSI), proposed by the Centers for Disease Control and Prevention (CDC) in 1999, recommends that ‘If drainage is necessary, use a closed suction drain and remove the drain as soon as possible’ [1]. Recent randomized controlled trials and meta-analyses have supported the limited use of prophylactic intra-abdominal drainage for many gastrointestinal surgeries [2-4]. In gastric surgery, drain placement is designed for the removal of fluid collections or for the early detection of postoperative bleeding, pancreatic fistulas, anastomotic leakage, and intra-abdominal infections. Incorrect use of an intra-abdominal drain can cause exudation of protein-rich ascitic fluid, which may lead to hypovolemia and hypoproteinemia, or facilitate retrograde bacterial contamination. With recent advances in interventional radiology, image-guided percutaneous drainage and aspiration procedures after the onset of complications now entail a low risk of intestinal injury [5]. Unnecessary drain placement must thus be avoided, but little is known about the value of prophylactic drainage following laparoscopic distal gastrectomy (LDG). In addition, surgeons often feel the need to place an intra-abdominal drain based on their intraoperative impression regarding factors such as the degree of difficulty of the surgical procedure and/or the level of surgical completeness and/or their own surgical experience. In this study, we investigated the significance of prophylactic intra-abdominal drain placement after LDG for gastric cancer.

## Methods

### Patients

Seventy-eight consecutive patients with gastric cancer who underwent LDG with a curative intent between January 2011 and April 2014 in our department were retrospectively analyzed. All operations were performed by the same operative team and were completed laparoscopically. The patients were divided into two groups according to the placement of a prophylactic intra-abdominal drain following LDG. The ‘drain group’ comprised 45 patients who underwent LDG, from January 2011 to December 2012, with routine use of a prophylactic intra-abdominal drain. The ‘no-drain group’ comprised 33 patients who underwent LDG, from January 2013 to April 2014, without any intra-abdominal drain placement.

### Tumor staging

The ‘Japanese classification of gastric carcinoma: 3rd English edition’ was used for tumor staging [6]. The appropriate extent of gastrectomy and lymphadenectomy (D1+ or D2) was determined according to the recommendation of the ‘Japanese gastric cancer treatment guidelines 2010 (ver. 3)’ [7].

Clinical classification of tumor depth and nodal involvement was evaluated using preoperative barium radiography, upper gastrointestinal tract endoscopy, abdominal ultrasonography, computed tomography (CT), and endoscopic ultrasonography and was finally confirmed based on the pathologic findings.

### Indications for LDG

Eligibility criteria included histologically proven adenocarcinoma of the gastric middle body or lower body, tumor status lower than T4b, no distant metastasis, and no lymph nodes larger than 1 cm in the hepatoduodenal ligament or in the paraaortic area. Exclusion criteria included carcinoma in the remnant stomach, linitis plastica, and synchronous or metachronous double cancers in the previous 10 years.

### Surgical technique

The patient was placed in the supine position with legs apart, under a combination of epidural and general anesthesia. A 12-mm trocar was inserted by the open method in the umbilical region as a port for the camera. After the completion of pneumoperitonization with the carbon dioxide pressure at 8 mmHg, two additional 12-mm trocars were inserted in the right lower and left upper abdomen and two 5-mm trocars were placed in the left lower and right upper abdomen.

Mobilization of the stomach and D1+ or D2 lymph node dissection were carried out in the conventional manner. The stomach and duodenum were divided with an endoscopic linear stapler (Endo GIA Ultra Universal stapler; Covidien, Mansfield, MA, USA). Typically, a two-thirds to four-fifths distal gastrectomy was performed. The dissected stomach was externalized through the umbilical incision, which was extended to approximately 4 cm, and a side-to-side jejunojejunostomy was performed. Subsequently, an intracorporeal antecolic Roux-en Y reconstruction with an antiperistaltic gastrojejunostomy was completed.

In the drain group, a low-suction silicon drain (J-VAC: BLAKE Silicon Drain Kits; Ethicon, Inc., Somerville, NJ, USA) was placed in the foramen of Winslow via the right upper quadrant incision. The silicon drain has a solid-core structure with a three-dimensional core, which allows the lumen to retain its round shape, and four drainage channels inside the lumen. The suction bag has an internal spring structure, which provides constant and sustainable negative pressure.

### Postoperative management

Patients in both groups were managed in the same manner using a standardized postoperative clinical protocol. Prophylactic antibiotics were administered every 6 h for 24 h from the beginning of surgery. As a rule, oral intake of water was initiated on the first day following the surgery, and liquid meals were resumed on postoperative day 5. All patients underwent upper gastrointestinal series (UGIs) with water-soluble contrast medium after surgery, and anastomotic leakage was defined based on the findings of UGIs or CT studies. Intra-abdominal abscess was defined as demonstrable fluid collection on CT in patients with high-grade fever or elevated serum C-reactive protein levels.

In principle, the silicon drain was removed after postoperative UGIs confirmed the absence of anastomotic leakage.

### Assessment of surgical and postoperative outcomes

Surgical outcomes were evaluated in terms of extent of lymph node dissection, intraoperative blood loss, operation time, and requirement for blood transfusion.

Postoperative outcomes were evaluated in terms of postoperative days until the resumption of oral intake of fluids and food, time to first flatus and defecation, postoperative complications, and the length of the postoperative hospital stay. The postoperative complications comprised surgery-related local complications and systemic complications; the former included anastomotic or duodenal stump leakage, intra-abdominal abscess formation, wound infection, anastomotic edema, Roux-en Y stasis, and Petersen’s hernia, and the latter included pulmonary infection and cerebral infarction.

All postoperative complications were monitored according to the Clavien-Dindo classification within 4 weeks of surgery [8], and any event of severity greater than grade II was counted as a postoperative complication.

### Risk assessment for the occurrence of postoperative complications

To conduct the risk analysis, we assessed the impacts of operative time and intraoperative blood loss on the occurrence of postoperative complications, particularly surgery-related postoperative local complications.

### Statistical analysis

Statistical analysis was performed using JMP statistical software (SAS Institute Inc., Cary, NC, USA). Values are expressed as the mean ± standard deviation (SD). Comparisons between the two study groups were performed using Student’s *t*-test, the chi-square test, the Mann-Whitney *U*-test, or Fisher’s exact test as appropriate. For all tests, a *p* value less than 0.05 was considered statistically significant.

Risk factors for the occurrence of postoperative complications were analyzed in relation to patients’ preoperative conditions and intraoperative factors by logistic regression analysis.

Permission to perform this retrospective study was obtained from the ethical board of our institution.

## Results

### Characteristics of the patients

Forty-five and thirty-three patients were enrolled in the drain group and the no-drain group, respectively (Table 1). There were no significant differences in terms of the mean age of the patients, male/female ratio, body mass index, and concurrent diseases between the drain group and the no-drain group. In addition, there were no significant differences in the tumor location, tumor diameter, depth of the tumor, nodal metastasis, and tumor stage between the two groups.

**Table 1:** **Characteristics of the patients**

		**Drainage group**	**No-drainage group**	
		**(** ***n*** **= 45)**	**(** ***n*** **= 33)**	***p*** **value**
Male:female		28:17	23:10	0.493a
Age (years)		70.5 ± 13.4	74.8 ± 9.8	0.125b
BMI (kg/m^2^)		23.2 ± 4.0	23.2 ± 4.0	23.2 ± 4.0
Concurrent illness				
None		7(15.6%)	7(21.2%)	0.520a
Arrhythmia		2(3.6%)	5(15.2%)	
Ischemic heart disease		7(12.7%)	2(6.1%)	
Hypertension		22(40.0%)	12(36.4%)	
Diabetes mellitus		6(10.1%)	5(15.2%)	
Cerebral infarction		4(7.3%)	4(12.1%)	
COPD		9(16.4%)	6(18.2%)	
Hyperlipidemia		9(16.4%)	3(9.1%)	
Other		7(12.7%)	5(15.2%)	
Location of tumor (M/L)		26/19	14/19	0.180a
Diameter of tumor (mm)		44.5 ± 31.6	39.0 ± 19.3	0.382b
Depth of tumor invasion	1a	11(24.4%)	5(15.2%)	0.177a
	1b	13(28.9%)	12(36.4%)	
	2	6(13.3%)	8(24.2%)	
	3	10(22.2%)	2(6.1%)	
	4a	5(11.1%)	6(18.2%)	
Lymph node metastasis	0	33(73.3%)	23(69.7%)	0.132a
	1	2(4.4%)	6(18.2%)	
	2	5(11.1%)	1(3.0%)	
	3a	5(11.1%)	2(6.1%)	
	3b	0(0%)	1(3.0%)	
Tumor stage	1a	24(53.3%)	14(42.4%)	0.245a
	1b	4(8.9%)	9(27.3%)	
	2a	5(11.1%)	1(3.0%)	
	2b	2(4.4%)	4(12.1%)	
	3a	4(8.9%)	2(6.1%)	
	3b	4(8.9%)	2(6.1%)	
	3c	2(4.4%)	1(3.0%)	

BMI body mass index, COPD chronic obstructive pulmonary disease, M middle, L lower, a chi-square test, b Student’s *t*-test.

### Operative data by group

All patients in each group were successfully treated with R0 surgery, and no patient required conversion to open surgery. Surgical outcomes are summarized in Table 2. Surgery-related factors, including lymph node dissection and operative time, were similar in the drain group and the no-drain group. The no-drain group was associated with less intraoperative blood loss compared to the drain group, but the difference was not statistically significant. Eight patients in the drain group received blood transfusions, while two patients in the no-drain group required blood transfusions.

**Table 2:** **Operative data per group**

		**Drain group**	**No-drain group**
		**(** ***n*** **= 45)**	**(** ***n*** **= 33)**
Lymph node dissection			
	D1+	25(55.6%)	16(48.5%)
	D2	20(44.4%)	17(51.5%)
Intraoperative blood loss (ml)		52.3 ± 59.9	40.8 ± 85.7
Operation time (min)		408.5 ± 84.5	413.2 ± 65.7
Blood transfusion		8(17.8%)	2(6.1%)

### Postoperative outcomes

Postoperative outcomes are summarized in Table 3. There was no significant difference in the mean postoperative time interval to first flatus and defecation, whereas the time to first oral intake of fluids and food was shorter in the no-drain group (first intake of fluids, *p* < 0.0001; first intake of food, *p* = 0.003). However, this result seemed to be due to a minor change in our postoperative clinical pathway in the middle of this study in January 2013.

**Table 3:** **Postoperative outcomes**

		**Drain group**	**No-drain group**	
		**(** ***n*** **= 45)**	**(** ***n*** **= 33)**	***p*** **value**
First drinking, POD		3.6 ± 3.1	1.0 ± 0.2	<0.0001a
First eating, POD		6.6 ± 3.2	4.8 ± 1.2	0.002a
First flatus, POD		2.2 ± 1.6	1.8 ± 0.9	0.136b
First defecation, POD		4.6 ± 2.3	3.8 ± 1.5	0.069b
Postoperative complication		14(31.1%)	10(30.3%)	0.892c
Surgery-related local complication				
Stump leakage		0(0%)	2(6.1%)	
Abscess		3(6.7%)	1(3.0%)	
Wound infection		4(8.9%)	1(3.0%)	
Anastomotic edema		3(6.7%)	1(3.0%)	
Stasis		4(8.9%)	2(6.1%)	
Petersen’s hernia		0(0%)	1(3.0%)	
Systemic complication				
Pulmonary infection		2(4.4%)	1(3.0%)	
Cerebral infarction		0(0%)	1(3.0%)	
Postoperative hospital stay (days)		16.4 ± 8.1	16.5 ± 8.1	0.982b
Complication	(-)	13.2 ± 3.4(*n* = 31)	12.3 ± 2.5(*n* = 23)	0.289b
	(+)	24.5 ± 10.5(*n* = 14)	26.1 ± 8.6(*n* = 10)	0.693b
Local complication	(-)	13.5 ± 3.4(*n* = 34)	13.3 ± 4.2(*n* = 25)	0.941b
	(+)	26.0 ± 10.7(*n* = 11)	26.5 ± 9.6(*n* = 8)	0.917b

POD postoperative day, a Mann-Whitney *U*-test, b Student’s *t*-test, c Chi-square test.

Overall postoperative complications based on the Clavien-Dindo classification were recognized in 31.1% and 30.3% of patients in the drain group and the no-drain group, respectively, and there was no significant difference in the rate of complications between the two groups (*p* = 0.892). There was no hospital mortality in this study, but one patient in the no-drain group who developed Petersen’s hernia required reoperation on postoperative day 8.

Regarding the postoperative hospital stay, no statistically significant difference was found between the drain group and the no-drain group. When taking the presence and absence of postoperative complications into consideration, the length of postoperative hospital stay did not differ between the two groups.

### Risk assessment for the occurrence of postoperative complications

Risk factors for the occurrence of postoperative complications were analyzed in relation to patients’ preoperative conditions and intraoperative factors, but no useful predictors of postoperative complications were identified (data not shown).

A comparison of operative time between patients with and without postoperative complications revealed that the patients with postoperative complications tended to have longer operative times than those without postoperative complications, but the difference was not significant (*p* = 0.121) (Table 4). Operative time was also compared between patients with and without postoperative complications in each group. No difference was found in the drain group, but patients who experienced postoperative complications in the no-drain group tended to have longer operative times than those without postoperative complications. However, the difference was not significant (*p* = 0.071).

**Table 4:** **Risk assessment for the occurrence of postoperative complication**

	**Postoperative complication**		
	**(-)**	**(+)**	***p*** **value**
Operation time (min)			
All cases	401.8 ± 79.2(*n* = 55)	431.3 ± 67.3(*n* = 23)	0.121a
Drain group (*n* = 45)	403.3 ± 88.1(*n* = 31)	421.3 ± 76.5(*n* = 14)	0.524a
No-drain group (*n* = 33)	399.6 ± 66.7(*n* = 23)	444.4 ± 54.2(*n* = 10)	0.071a
Intraoperative blood loss (ml)			
All cases	38.2 ± 51.0(*n* = 55)	69.6 ± 104.3(*n* = 23)	0.038a
Drain group (*n* = 45)	47.3 ± 55.8(*n* = 31)	64.6 ± 69.7(*n* = 14)	0.386a
No-drain group (*n* = 33)	25.4 ± 41.2(*n* = 23)	76.0 ± 141.4(*n* = 10)	0.121a

a Student’s *t*-test.

A comparison of the amount of intraoperative blood loss between patients with and without postoperative complications revealed that patients who experienced postoperative complications had a significantly larger amount of blood loss than those without postoperative complications (*p* = 0.038). The same tendency was found when the volume of intraoperative blood loss was compared between patients with and without postoperative complications in each group, but the differences were not significant (Table 4).

### Risk assessment for the occurrence of surgery-related postoperative local complications

A comparison of operative times between patients with and without surgery-related postoperative local complications revealed that patients who experienced surgery-related postoperative local complications had a significantly longer operative time than those without surgery-related postoperative local complications (*p* = 0.041) (Table 5). Analysis of operative times in each group revealed that patients with surgery-related postoperative local complications had a significantly longer operative time than those without surgery-related postoperative local complications in the no-drain group (*p* = 0.049).

**Table 5:** **Risk assessment for the occurrence of surgery-related postoperative local complication**

	**Surgery-related local complication**		
	**(-)**	**(+)**	***p*** **value**
Operation time(min)			
All cases	402.0 ± 77.6(*n* = 59)	436.9 ± 69.0(*n* = 19)	0.041a
Drain group (*n* = 45)	401.6 ± 85.7(*n* = 33)	429.9 ± 80.6(*n* = 12)	0.340a
No-drain group (*n* = 33)	402.5 ± 66.8(*n* = 25)	446.6 ± 52.9(*n* = 8)	0.049a
Intraoperative blood loss(ml)			
All cases	44.2 ± 61.2(*n* = 59)	57.4 ± 98.8(*n* = 19)	0.490a
Drain group (*n* = 45)	50.7 ± 55.8(*n* = 33)	57.3 ± 73.7(*n* = 12)	0.756a
No-drain group (*n* = 33)	35.4 ± 68.0(*n* = 25)	57.5 ± 131.7(*n* = 8)	0.534a

a Student’s *t*-test.

A comparison of the amount of intraoperative blood loss between patients with and without surgery-related postoperative local complications revealed that patients who experienced surgery-related postoperative local complications had a larger amount of blood loss than those without surgery-related postoperative local complications, but the difference was not significant (*p* = 0.490).

## Discussion

It is a well-known fact that the CDC guideline recommends a closed suction drain for the prevention of SSI [1], and this recommendation is based on the literature by Moro *et al*. which reported that leaving an open drain in place for 3 days or longer is a risk factor for SSI [9,10]. As the microbial biomass in drains is reported to increase with time, even in a closed drain, it is important to remove the drain as soon as possible [9]. However, applying the CDC guideline to gastrointestinal surgery may entail some problems because the abovementioned studies were restricted to cardiovascular surgery with clean or Class 1 wounds, which differs from gastrointestinal surgery in terms of the situation regarding drain placement. In other words, it is questionable whether it would be appropriate to apply the same standard for these two different surgeries, that is, clean and clean-contaminated operations.

Laparoscopy-assisted distal gastrectomy, first introduced in 1991, has now been standardized and stabilized with safety [11]. Conventionally, an intra-abdominal drain has been routinely placed following gastrectomy for the purpose of early detection of postoperative bleeding, anastomotic leakage, and intra-abdominal infections. Although the routine use of a drain or catheter is considered to be unnecessary from the perspective of recent Enhanced Recovery After Surgery (ERAS) guidelines, no high-quality evidence exists regarding whether an intra-abdominal drain would prevent and alleviate postoperative complications after laparoscopy-assisted distal gastrectomy. Ishikawa *et al*. reported that routine prophylactic abdominal drainage following laparoscopy-assisted distal gastrectomy for early gastric cancer may not be necessary [12]. Furthermore, Albanopoulos *et al*. reported that placement of intra-abdominal drains after laparoscopic sleeve gastrectomy does not facilitate detection of leaks and abscesses [13]. Such complications can be diagnosed by clinical and radiological findings. Thus, our department performed LDG without any intra-abdominal drain placement from January 2013. Because little information is available on routine prophylactic drainage after LDG [14,15], this study investigated the influence of the presence or absence of prophylactic intra-abdominal drain placement on the postoperative outcomes in patients who underwent LDG for gastric cancer.

Although postoperative complications were recognized in about 30% of patients in both the drain group and the no-drain group in the current study, no anastomotic leakage was observed for either gastrojejunostomy or jejunojejunostomy after LDG. Anastomotic leakage following gastrectomy, which requires early detection and subsequent appropriate measures, is a rare type of postoperative complication with a rate of occurrence of around 1% [16]. Moreover, we could not confirm any advantage associated with prophylactic drain placement in relation to anastomotic insufficiency after LDG. Meanwhile, intra-abdominal abscess and SSI occurred more frequently in the drain group, suggesting that retrograde infections through the drain or drainage failure in cases with pancreatic fistulas may be involved in such infectious complications. Among three patients who developed intra-abdominal abscesses in the drain group, only one patient required interventional radiology for abscess drainage, while the other two patients were successfully managed with antibiotics and fasting. Only one patient who developed an intra-abdominal abscess in the no-drain group was intensively treated with broad-spectrum antibiotics and fasting. Administration of broad-spectrum antibiotics could be a good conservative management strategy. Based on these facts, prophylactic drainage following LDG may not always be practical for the reduction of postoperative surgery-related postoperative local complications.

Placement of a prophylactic drain following LDG had no impact on the length of the postoperative hospital stay in this study. Among the patients who experienced postoperative complications, however, we found that patients in the drain group tended to have a shorter postoperative hospital stay than those in the no-drain group. This finding suggests the possibility that drainage would be effective in patients who experienced surgery-related postoperative local complications.

Predicting the risk factors for postoperative complications after LDG by utilizing patients’ preoperative conditions was not successful in this study. On the other hand, intraoperative factors such as the operative time and the amount of intraoperative blood loss affected the occurrence of postoperative complications following LDG. The postoperative risk assessment should thus include intraoperative factors as well as patients’ preoperative conditions. A prophylactic drain may be useful in patients at higher risk, for example, patients with a longer operative time or massive intraoperative bleeding. At present, placement of a prophylactic drain should be decided with consideration of hospital volume, surgeon volume, skill of the individual surgeon, risks of postoperative complications, and a backup system when complications occur. Prospective randomized studies with large sample sizes should be conducted to evaluate the significance of prophylactic intra-abdominal drains following LDG.

## Conclusions

A prophylactic drain may be useful in patients at higher risk and in those with a longer operative time or massive intraoperative bleeding.
